# The acute transcriptional response to resistance exercise: impact of age and contraction mode

**DOI:** 10.18632/aging.101904

**Published:** 2019-04-15

**Authors:** Colleen S. Deane, Ryan M. Ames, Bethan E. Phillips, Michael N. Weedon, Craig R.G. Willis, Catherine Boereboom, Haitham Abdulla, Syed S.I. Bukhari, Jonathan N. Lund, John P. Williams, Daniel J. Wilkinson, Kenneth Smith, Iain J. Gallagher, Fawzi Kadi, Nathaniel J. Szewczyk, Philip J. Atherton, Timothy Etheridge

**Affiliations:** 1 Department of Sport and Health Sciences, College of Life and Environmental Sciences, University of Exeter, Exeter EX1 2LU, UK; 2 Living Systems Institute, University of Exeter, Exeter EX4 4QD, UK; 3 MRC-ARUK Centre of Research Excellence and National Institute of Health Research, Biomedical Research Centre, Postgraduate Entry Medical School, Royal Derby Hospital Centre, School of Medicine, University of Nottingham, Derby DE22 3DT, UK; 4 Genetics of Complex Traits, University of Exeter Medical School, University of Exeter, Exeter EX1 2LU, UK; 5 Department of Surgery, Postgraduate Entry Medical School, Royal Derby Hospital Centre, School of Medicine, University of Nottingham, Derby DE22 3DT, UK; 6 Faculty of Health Sciences and Sport, University of Stirling, Stirling FK9 4LA, UK; 7 School of Health Sciences, Örebro University, Örebro 70182, Sweden; * Equal contribution; # Joint senior authorship

**Keywords:** concentric, eccentric, sarcopenia, transcriptome

## Abstract

Optimization of resistance exercise (RE) remains a hotbed of research for muscle building and maintenance. However, the interactions between the contractile components of RE (i.e. concentric (CON) and eccentric (ECC)) and age, are poorly defined. We used transcriptomics to compare age-related molecular responses to acute CON and ECC exercise. Eight young (21±1 y) and eight older (70±1 y) exercise-naïve male volunteers had *vastus lateralis* biopsies collected at baseline and 5 h post unilateral CON and contralateral ECC exercise. RNA was subjected to next-generation sequencing and differentially expressed (DE) genes tested for pathway enrichment using Gene Ontology (GO). The young transcriptional response to CON and ECC was highly similar and older adults displayed moderate contraction-specific profiles, with no GO enrichment. Age-specific responses to ECC revealed 104 DE genes unique to young, and 170 DE genes in older muscle, with no GO enrichment. Following CON, 15 DE genes were young muscle-specific, whereas older muscle uniquely expressed 147 up-regulated genes enriched for cell adhesion and blood vessel development, and 28 down-regulated genes involved in mitochondrial respiration, amino acid and lipid metabolism. Thus, older age is associated with contraction-specific regulation often without clear functional relevance, perhaps reflecting a degree of stochastic age-related dysregulation.

## Introduction

Maintaining a healthy skeletal muscle mass across the lifecourse is essential to preserve locomotory function and whole-body metabolic health [1,2]. Accordingly, progressive muscle wasting such as with ageing (‘sarcopenia’) is associated with increased risk of falls, metabolic diseases and mortality [3,4]. Resistance exercise (RE) training (RET) represents the most effective lifestyle intervention for increasing/maintaining muscle mass and function across the lifespan [5]. One reason it is so important to optimise RET-strategies for maximising muscle mass gains in older age is because older adults exhibit blunted protein synthetic [6,7] and hypertrophic [8] responses to RE/T (commonly termed *anabolic resistance*). There thus remains a significant need to optimise anabolic responses to RE in older adults and to understand the molecular basis of any adaptive deficits.

Conventional RE involves both shortening (concentric, CON) and lengthening (eccentric, ECC) contractions, which induce distinct architectural adaptations. Indeed, adaptations to CON favour increases in pennation angle while ECC favours increases in fascicle length [9,10]. Chronically, ECC RET may also lead to greater muscle hypertrophy and strength gains versus CON RET [11]. As such, it is being increasingly claimed that ECC-type RET represents an intervention with potential to facilitate muscle maintenance in ageing [12]. Nonetheless, of the limited studies comparing adaptations to conventional RET (i.e. CON+ECC) versus ECC in older adults, conflicting results suggest that isolated ECC either augments [13,14] or confers no additional benefits [15–17] in total muscle mass and/or isometric leg strength of older adults (versus CON or conventional RET).

Transcriptomic responses to acute ECC and CON exercise have also demonstrated specific molecular regulation. For example, in healthy young adults, combined ECC+CON (versus CON alone) uniquely upregulated 28 genes involved in ontologies relating to inflammatory processes, energy metabolism and extracellular matrix function, with no apparent downregulated genes [18]. In a second study, up to 24 h post-exercise, ECC versus CON differentially regulated 51 transcripts involved in processes of protein synthesis, stress responses and sarcolemmal structure [19]. However, just 8 and 11 genes were uniquely regulated by ECC at 3 and 6 h post-exercise, respectively [19]. Transcriptional responses to ECC/CON have not been directly compared in older adults. Nonetheless, 4 h after traditional RE loading (i.e. combined ECC+CON) older versus younger muscle displayed 595 differentially regulated transcripts associated with various signalling processes [20]. Similarly, 318 “age-specific” genes were uniquely expressed 24 h post-RE, enriched for inflammatory, immune system, protein degradation and stress response-related pathways [21]. Despite providing seminal insight, these transcriptomic studies have limitations including i) lack of baseline comparison [18], ii) variable post-exercise time frames (4-8 h [18],), iii) inability to distinguish ECC effects from CON [18], iv) low subject N (n=3 [18,19]), v) inconsistency between ECC- and CON-unique transcriptional profiles precluding alignment of molecular responses and contraction mode. Furthermore, despite a lack of coherence in differentially expressed genes between studies (at least as a factor of age [22]) ageing could be associated with altered transcriptional responses to exercise that could contribute to poorer adaptation of ageing muscle to RET.

Given maladaptation to exercise (RE/RET) in ageing, and the distinct metabolic and molecular responses to contraction mode, we examined the acute transcriptional response to isolated CON and isolated ECC contractions, in young and older adults. We hypothesised that there would be age-related differences in the transcriptional response to exercise that may help explain age-related differences in the adaptive responses to exercise.

## RESULTS

### Sequence quality and removal of outliers

FastQC was used to judge the quality of the sequence data. As samples contained no over-represented sequences or adapter sequences and the illumina base quality scores were always >30, no filtering or trimming was performed. A multi-dimensional scaling (MDS) plot to characterise the variation between samples identified the presence of two outliers in the samples (Figure S1A). The samples, a young baseline sample and an older 5 h post-ECC exercise sample, were removed from all subsequent analysis. The MDS plot of the remaining data showed a more clustered set of samples with no obvious outliers (Figure S1B).

### Age-independent muscle transcriptomic responses to CON and ECC exercise

**Figure 1:**
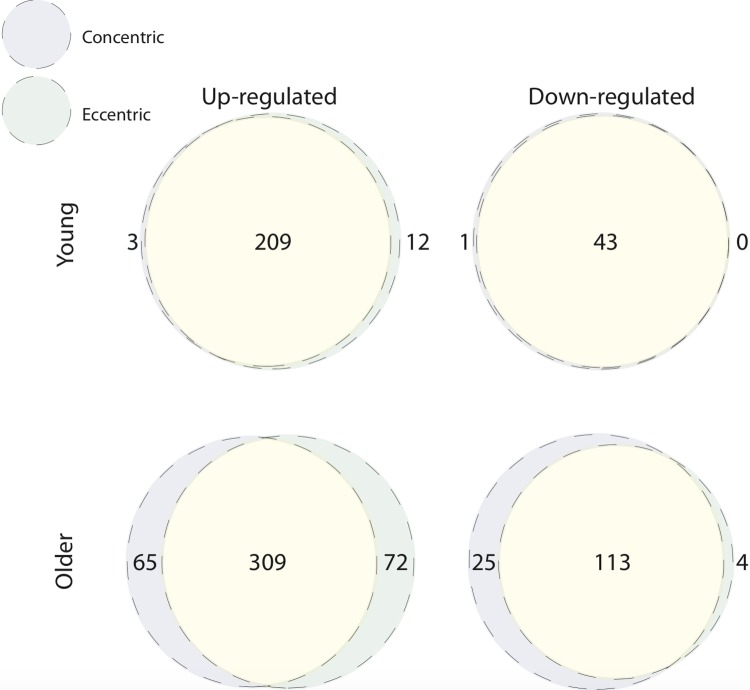
**Concentric and eccentric exercise induce a common transcriptomic response in young and older muscle.** Overlap of contraction mode-associated differentially expressed genes within age groups. Near complete overlap exists between up-regulated (top left Venn) and down-regulated (top right Venn) genes, 5 h after concentric and eccentric exercise in young muscle. Up-regulated (bottom left Venn) and down-regulated (bottom right Venn) genes in older muscle after concentric and eccentric exercise also display predominant overlap.

**Figure 2:**
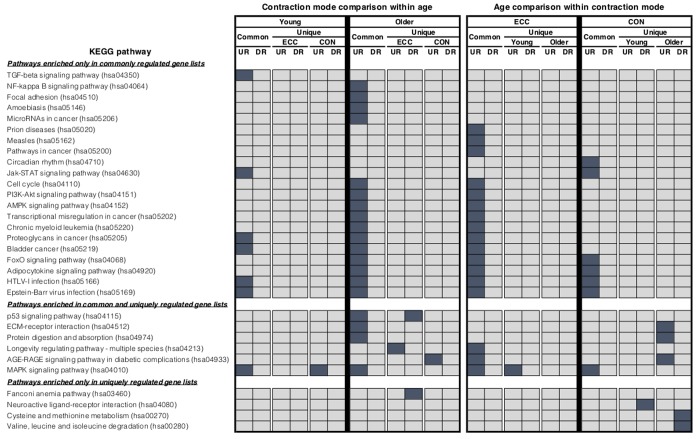
**KEGG pathway analysis.** Summary of significantly enriched KEGG pathways for the lists of differentially expressed genes that were identified as being either commonly or uniquely regulated when comparing between contraction mode within age group and between age groups within each contraction mode using the rank-rank hypergeometric overlap. Dark blue shading highlights which KEGG terms were found to be significantly enriched (adjusted *P*-value <0.05) in each condition.

Comparing baseline to 5 h post-CON and -ECC exercise, the muscle transcriptome displayed a pattern of significant differential gene expression in both age groups (Table 1). We next directly compared these differentially expressed genes between post-CON and post-ECC exercise, within age groups (Figure 1). In young adults the up- and down-regulated transcriptional profiles of CON and ECC contractions are highly similar (209 and 43 commonly up- and down-regulated genes, respectively). However small contraction mode-specific differences exist, most notably with 12 up-regulated genes unique to ECC. Similarly, in older adults there is large overlap between CON and ECC differentially expressed genes (309 and 113 commonly up- and down-regulated genes, respectively), with enrichment in multiple KEGG pathways including the NF-κB, focal adhesion and energy-sensing pathways. Despite the higher number of contraction-unique up-regulated genes in older muscle (Figure 1), these gene sets show no enrichment for gene ontology (GO) terms suggesting that the genes do not coherently represent any specific functions or that these sets comprise poorly annotated genes. However, KEGG analysis showed ECC-specific upregulation of a longevity pathway, and CON-specific upregulation of the AGE-RAGE signalling pathway (Figure 2). Full lists of differentially expressed genes from baseline to post-exercise, and for the overlap between contraction types can be found in Table S1. Complete lists of enriched GO terms and KEGG pathways for all contraction mode-associated differentially expressed genes can be found in Table S2 and Table S3, respectively.

**Table 1:** Total numbers of significantly differentially expressed genes 5 h after concentric and eccentric exercise, in skeletal muscle of young and older individuals.

	**Concentric exercise**			**Eccentric exercise**	
	**Up-regulated**	**Down-regulated**		**Up-regulated**	**Down-regulated**
**Young**	227		63	590	204
**Older**	665		328	724	198

### The effect of age on the muscle transcriptomic response to CON and ECC exercise

**Figure 3:**
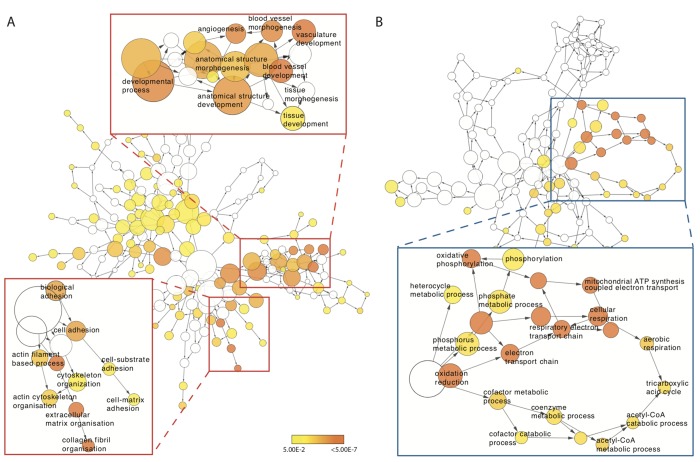
**Concentric exercise-mediated transcriptomic profile of older muscle.** (**A**) GO terms enriched for genes in older muscle that display increased expression 5 h post-concentric exercise. Red inserts highlight increased expression of GO terms associated with cytoskeletal, cell adhesion and extra-cellular matrix (bottom right) and blood vessel development (top left). (**B**) GO terms enriched for genes in older muscle that display reduced expression 5 h post-concentric exercise. Blue insert highlights reduced expression of GO term network associated with mitochondrial metabolism. Each node in the network represents a GO term with the size of each node corresponding to the number of genes associated with that term. Significantly enriched terms are coloured yellow with more significant terms a deeper shade.

To determine whether ageing muscle displays a disparate post-exercise transcriptional profile compared to the young, GO term enrichment was first assessed for differentially expressed genes between baseline and post-CON and baseline and post-ECC within age groups. In the young, we identified between 6-115 GO terms in the ‘Biological Processes’ ontology in up- or down-regulated genes after CON and ECC exercise. Whilst more specific terms such as ‘regulation of TOR signalling cascade’ were expectedly identified, more predominant (e.g. common to both CON and ECC responses) were high-level GO terms including ‘signalling,’ ‘multicellular organismal process’ and ‘developmental process’, which are too broad to be informative. Thus, no consistent functional pattern emerges for the post-exercise transcriptional response in young muscle. The older muscle response to ECC similarly identified numerous high-level GO terms enriched in up-regulated (138) and down-regulated (39) genes. Full lists of these GO terms can be viewed in Table S2. However, a clear post-CON specific pattern of GO term enrichment arose in older muscle: terms associated with blood vessel development and cell adhesion were enriched in up-regulated genes, and several terms relating to mitochondrial respiration were enriched in the set of down-regulated genes (Figure 3).

**Figure 4:**
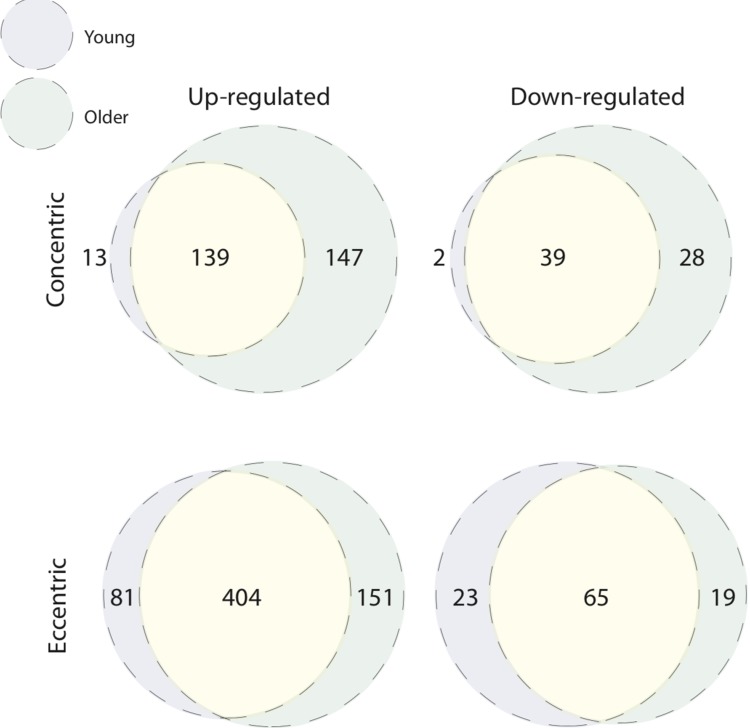
**Unique age-related transcriptomic response to concentric and eccentric exercise.** Overlap of age-associated differentially expressed genes within contraction modes. Both young and older muscle display unique signatures of differential gene expression 5 h post-concentric (up-regulated, top left Venn; down-regulated, top right Venn) and post-eccentric (up-regulated, bottom left Venn; down-regulated, bottom right Venn) exercise.

**Figure 5:**
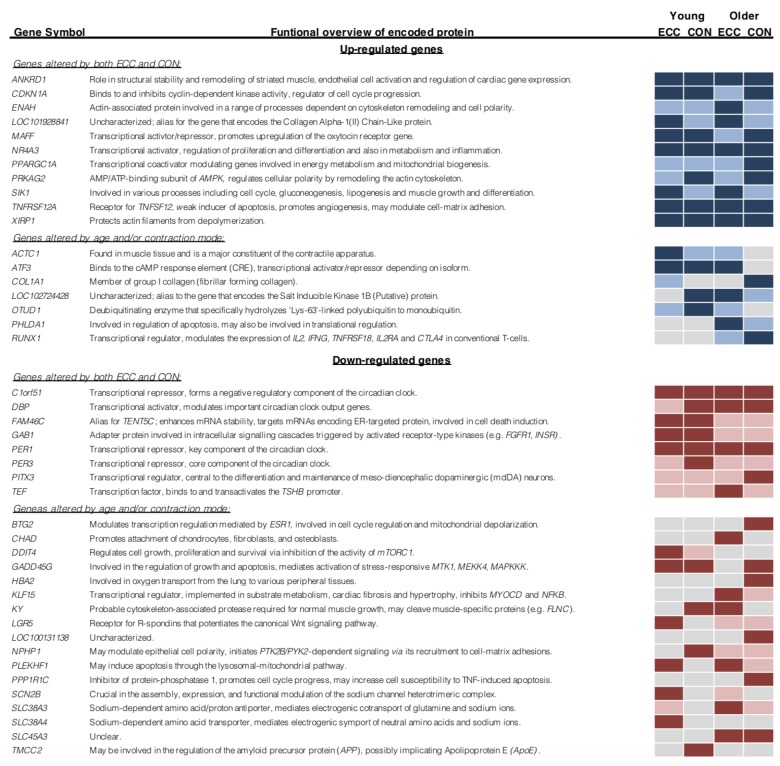
**Top 10 ranked differentially expressed genes, classified by contraction mode and age group.** Light blue/ red shading denotes gene differential expression for that condition (*P*<0.05). Dark blue/ red shading indicates gene falls within the top 10 log-fold change ranked differentially expressed genes for that condition.

We next overlaid post-CON and post–ECC differentially expressed genes between young and older age groups to directly compare age-specific transcriptomic responses (Figure 4). After CON exercise, very few genes were uniquely up-regulated (13 genes) and down-regulated (2 genes) in young muscle, with no clear GO pattern. In contrast (and in line with older baseline to 5 h post-CON expression changes), post-CON older muscle displayed: i) unique up-regulation of 147 genes enriched for terms associated with cell adhesion, extracellular organisation and blood vessel development and, ii) unique down-regulation of 28 genes enriched for terms relating to the metabolism and/ or catabolism of amino acids, lipids, carboxylic acid and DNA (Table 2). KEGG analysis further confirms GO findings that in older muscle, CON exercise specifically down-regulates pathways of amino acid metabolism, and up-regulates extra-cellular matrix-receptor related pathways (Figure 2). Despite comparably high numbers of uniquely up-regulated (151) and down-regulated (19) genes after ECC in the older group, and a differential ECC expression profile in the young (81 up-regulated and 23 down-regulated genes), there was no GO term enrichment for any age-dependent ECC gene sets. Complete lists of enriched GO terms for post-exercise differentially expressed genes between age groups can be viewed in Table S2. Due to the tendency for poor GO term enrichment, we further assessed the top 10 ranked DE genes per condition based on logarithmic fold change in expression to provide a functional overview of the most significantly altered genes (Figure 5), and assessed gene enrichment in KEGG pathways (Figure 2). However, KEGG analysis also failed to find ECC-specific gene enrichment in any biological pathway.

**Table 2:** GO terms enriched for significantly differentially expressed genes in older muscle, 5 h post-concentric exercise. Dashed lines separate broad functional GO term classes.

**GO-ID**	**GO description**	**Observed DE genes**	**Total genes in GO-ID**	***P*-raw**	***P*-corrected**
Up-regulated post-concentric exercise in older muscle only:					
7155	Cell adhesion	14	355	2.12E-04	3.24E-02
22610	Biological adhesion	14	355	2.12E-04	3.24E-02
43062	Extracellular structure organization	9	96	4.18E-06	1.71E-03
30198	Extracellular matrix organization	8	72	4.08E-06	1.71E-03
30199	Collagen fibril organization	6	22	2.81E-07	3.44E-04
1568	Blood vessel development	12	210	1.83E-05	4.47E-03
1944	Vasculature development	12	215	2.31E-05	4.71E-03
43588	Skin development	5	21	6.14E-06	1.88E-03

Down-regulated post-concentric exercise in older muscle only:					
9081	Branched chain family amino acid metabolic process	2	16	5.24E-04	3.10E-02
9082	Branched chain family amino acid biosynthetic process	1	2	4.33E-03	4.60E-02
9098	Leucine biosynthetic process	1	2	4.33E-03	4.60E-02
19482	Beta-alanine metabolic process	1	1	2.17E-03	4.60E-02
9063	Cellular amino acid catabolic process	2	42	3.64E-03	4.60E-02
9083	Branched chain family amino acid catabolic process	2	12	2.90E-04	2.86E-02
6550	Isoleucine catabolic process	1	2	4.33E-03	4.60E-02
19484	Beta-alanine catabolic process	1	1	2.17E-03	4.60E-02
6574	Valine catabolic process	1	1	2.17E-03	4.60E-02
34440	Lipid oxidation	2	34	2.39E.03	4.60E-02
19395	Fatty acid oxidation	2	34	2.39E-03	4.60E-02
6635	Fatty acid beta-oxidation	2	29	1.74E-03	4.60E-02
9062	Fatty acid catabolic process	2	37	2.83E-03	4.60E-02
46395	Carboxylic acid catabolic process	4	80	2.21E-05	3.27E-03
42732	D-xylose metabolic process	1	1	2.17E-03	4.60E-02
5997	Xylulose metabolic process	1	1	2.17E-03	4.60E-02
45072	Regulation of interferon-gamma biosynthetic process	1	2	4.33E-03	4.60E-02
45078	Positive regulation of interferon-gamma biosynthetic process	1	2	4.33E-03	4.60E-02
46113	Nucleobase catabolic process	1	2	4.33E-03	4.60E-02
6208	Pyrimidine base catabolic process	1	2	4.33E-03	4.60E-02
19859	Thymine metabolic process	1	2	4.33E-03	4.60E-02
6210	Thymine catabolic process	1	2	4.33E-03	4.60E-02
9439	Cyanate metabolic process	1	2	4.33E-03	4.60E-02
9440	Cyanate catabolic process	1	2	4.33E-03	4.60E-02
6082	Organic acid metabolic process	5	371	9.19E-04	4.53E-02
16054	Organic acid catabolic process	4	80	2.21E-05	3.27E-03
44282	Small molecule catabolic process	4	179	5.06E-04	3.10E-02
44270	Cellular nitrogen compound catabolic process	2	46	4.35E-03	4.60E-02

## DISCUSSION

Loading muscle via CON or ECC RET induce divergent architectural and functional adaptations. The role of contraction mode on acute responses of ageing muscle to RE is poorly understood. Here we report that minor transcriptional differences characterise isolated ECC (versus isolated CON) in young muscle and that this pattern persists in older muscle. However, larger differentially expressed gene sets unique to age were observed for both ECC and CON. Whilst ECC-unique genes in older adults failed to cluster into any functional categories, the ageing CON profile was enriched for up-regulated cell adhesion, extracellular organisation and blood vessel development pathways, and down-regulation of genes associated with mitochondrial respiration, amino acid and lipid metabolism, and DNA regulation.

### Small differences characterise transcriptional responses to CON versus ECC

In spite of well-established acute and chronic adaptive differences, in young sedentary, healthy muscle we report overlap in the majority of genes acutely responsive to both CON and ECC. The notable exception of 12 up-regulated genes unique to ECC suggests limited contraction mode-specific transcriptional sensitivity, and these failed to cluster to ontology groups. This corroborates previous reports that of 3,300 transcripts examined, 11 genes were uniquely differentially regulated 6 h post-ECC, which did not associate with any functional classifications [19]. Similarly, Chen and colleagues [18] found 28 up-regulated and 0 down-regulated genes (of between 625-1,125 post-exercise differentially expressed genes) 4-8 h after ECC+CON versus CON alone. Thus, irrespective of contraction mode, unaccustomed exercise *per se* associates with a somewhat “generic” acute global genomic response. It is, however, plausible that transcriptional differences emerge over repeated bouts of RE, as muscle develops phenotypic specificity to ECC and CON. Interestingly, although the pattern of large transcriptional overlap remained, older muscle displayed a higher number of ECC and CON specific differentially expressed genes. Nonetheless, these failed to cluster to any gene GO terms, thus the functional meaning of this slightly more pronounced contraction mode-related difference is unclear. Whilst individual consideration of all CON- and ECC-unique genes is out of scope herein, future detailed analysis of this gene set might provide new mechanistic insight into poor ageing muscle adaptation to exercise.

We further compared our list of contraction mode specific DE genes with those identified in previous microarray studies [18,19,23] and found little overlap (with the exception of USP2 and NR4A1 [23] for young muscle ECC response, and LPP [18] and TM4SF1 [23] for older muscle ECC response). Despite lack of direct alignment in DE genes across human studies in young muscle - in all studies, increased expression of heat shock proteins (HSP) is consistently identified as a post-ECC signature (HSPB1, HSPB8, CRYAB [19], HSPA1B, DNAJB4 [18], DNAJB2 [23] and DNAJB1 presently). Whilst we identified other HSP’s as commonly up-regulated by both ECC and CON (Table S1), ECC appears to associate with an acute HSP response superseding that observed after CON. HSP’s are responsive to environmental stress and serve to stabilise proteins during cellular damage by facilitating protein folding during periods of increased protein synthesis, and saving ‘damaged’ proteins from further unfolding and trafficking to proteolytic machinery for degradation [24]. As such, HSP activation might occur as a post-translational, satellite cell-independent regenerative response to the cellular disruption caused by ECC contractions and the additional (i.e. beyond that required by CON) need for new proteins to replace and repair areas of localised muscle damage.

### Age modifies the post-CON and -ECC muscle transcriptome

ECC exercise altered the expression of numerous genes (total 170) uniquely in older muscle versus the young. It is however curious that none of the ECC responsive older muscle-specific genes clustered to any gene ontology terms. For example, no age-specific cell adhesion GO pattern or KEGG pathway enrichment was observed after ECC alone (which, conversely, was found post-CON), where the greater mechanical strain might be anticipated to stimulate a significant mechano-transduction response. This lack of ontological enrichment implies a certain randomness to the ageing muscle transcriptional response to ECC. This might support the ‘disposable soma’ theory of ageing [25] which proposes cellular and molecular ageing as an inherently stochastic process. Such influence of chance events and/ or random responses to external cues could explain the large differences observed in the rate and onset of ageing even among isogenic populations of mice and nematodes [26,27]. Indeed, stochastic ageing might be particularly relevant to muscle, which displays early onset and profound defects across the lifecourse, e.g. versus neuronal decline [26]. Mechanical loads also direct the stochasticity of biochemical reactions [28]. The greater (versus CON) muscular mechanical strain imposed by ECC may thus promote a stochastic transcriptional response, as observed herein. The anticipated net result of such random post-exercise molecular events would be loss of adaptive specificity, such as appropriate sarcomere remodelling and/ or cumulative muscle deterioration. Since these are known sarcopenic phenotypes, stochasticity might underpin at least part of poor muscle ageing.

Virtually all post-CON responsive genes in the young corresponded in older muscle, inferring age *per se*, does not reduce the transcriptional capacity to appropriately respond to CON. Nonetheless, older muscle displayed additional subsets of up- and down-regulated genes versus the young group. For example, the 147 genes specifically up-regulated in older muscle after CON were enriched for genes associated with blood vessel development and cell-cell adhesion GO terms, and altered cell adhesion pathways was further corroborated by KEGG pathway analysis. Interestingly, this represents an ontological opposite of the baseline ageing expression profile (data not shown) suggestive of a compensatory attempt of older muscle to correct age-related transcriptional abnormalities. Since this presumed corrective mechanism is ineffective at fully mitigating poor ageing muscle adaptation to exercise (i.e. the well-established ‘anabolic blunting’ phenomenon [6–8]), future investigation of the multifaceted role of impaired cell adhesion and associated mechanically-mediated intracellular signalling (e.g. via integrin-adhesome complexes) might provide new insight into the mechanisms of blunted ageing muscle exercise responses. Acute CON also associated with an age-specific down-regulation of certain metabolic pathways. Genes supressed post-CON in the older group clustered to a distinct subset of mitochondrial function, fatty acid and amino acid metabolism ontologies (the latter also confirmed by KEGG analysis). Diminished mitochondrial volume [29] and oxidative capacity [30–32] are frequently reported features of ageing muscle. Nonetheless, such studies are typically poorly controlled for confounders to confer age effects, i.e. physical activity levels [33].

The present study’s recruitment of sedentary young and older adults provides evidence that age *per se* can influence the muscle mitochondrial response to unaccustomed muscle contraction. Importantly, whilst aerobic and RE-training increase ageing muscle mitochondrial content and function [34], these adaptations remain below that of healthy young counterparts [35,36]. Our data provide preliminary evidence for a transcriptional basis of attenuated adaptation and implicates mitochondrial insensitivity to exercise (with CON at least) in the aetiology of ageing muscle deterioration. Finally, CON also down-regulated several amino acid metabolic and catabolic ontologies/ KEGG pathways, implying a post-CON disturbance in ageing muscles’ ability to process the substrates of muscle protein turnover. It is therefore possible that abnormal amino acid processing contributes to anabolic blunting of ageing muscle during exercise training [6–8].

## METHODS

### Subjects

Eight young (age, 21±1 y; body mass index, 23±2 kg/m^2^; 80% ECC 1RM, 211±14 kg; 80% CON 1RM, 122±11 kg) and eight older (age, 70±1y; body mass index, 26±1 kg/m^2^; 80% ECC 1RM, 155±1 kg; 80% CON 1RM, 79±6 kg) healthy male volunteers were recruited to this study. Because muscular adaptation occurs following a single bout of exercise to diminish the post-exercise regenerative response (i.e. the ‘repeated bout effect’ [37]), all recruited volunteers were exercise naïve, defined here as not participating in any form of structured exercise regime, and having no history of exercise training within the previous 12 months. Ethical approval was obtained from the University of Nottingham Medical School Ethics Committee, and all studies carried out in accordance with the Declaration of Helsinki. All volunteers were health-screened prior to participation.

## Study design

*Visit 1:* Volunteers were asked to refrain from exercise for 72 h and before testing performed an overnight fast with water consumed *ad libitum*. Volunteers arrived at ~9:00 h for a baseline muscle biopsy from the *m. vastus lateralis* of a randomised leg. Biopsies were taken under sterile conditions using local anaesthetic (1% lidocaine), blotted on gauze to remove excess blood, snap frozen in liquid nitrogen and stored at -80°C. *Visit 2:* After 96 h having conducted no exercise, volunteers performed an overnight fast and arrived at the laboratory at ~8.30 h after consuming a 250 ml liquid high energy nutritionally complete drink (Fortisip, Nutricia) at 07:00 h. Volunteers remained fasted thereafter until the study was complete at ~17.30 h. The isolated CON and isolated ECC knee extension exercise protocols were then performed; legs were randomised to unilateral CON-only and contralateral ECC-only exercise and the order in which CON and ECC were performed was also randomised. Exercise was performed on a modified leg press designed to permit either CON or ECC contractions in isolation within a single leg [9] with a starting knee angle of 90° for CON contractions and 180° for ECC contractions. For each isolated contraction mode, after an isolated CON/ECC warm-up consisting of 2 sets of 6 repetitions set at 40 kg volunteers, isolated CON/ECC one-repetition maximum (1-RM) was determined at a starting load of 50% estimated 1-RM, with subsequent attempts separated by 3 min and the resistance increased until a controlled contraction (~3 seconds) through the full range of motion could no longer be performed. Immediately after CON/ECC 1-RM had been established, the exercise protocol was performed comprising 7 sets of 10 repetitions (~3 second contractions) at 80% CON-specific or ECC-specific 1-RM, with 2-minute rest between sets. After ~1 h recovery the above exercise protocol was performed for the second contraction mode on the opposing leg. Finally, 5 h following the cessation of each exercise protocol a muscle biopsy was collected from the *m. vastus lateralis* of each leg.

### RNA sequencing

All RNA extraction, library preparations and next generation sequencing was performed by the Beijing Genomics Institute. Total RNA was isolated by using TRIzol reagent. Of the 8 volunteers, RNA integrity was low for 1 young and 1 older volunteer at all time points (i.e. baseline, 5 h post-ECC and 5 h post-CON) and were excluded from further analysis. Samples for 1 older volunteer (5 h post-ECC) and 2 young volunteers (5 h post-ECC and 5 h post-CON) also had low RNA integrity and were also excluded from analysis. Library preparations on the remaining 39 samples were performed using TruSeq RNA library preparation kits. Next generation sequencing was performed using the HiSeq 3000/HiSeq 4000 Illumina sequencing by synthesis chemistry systems, using advanced patterned flow cell technology for maximised level of sample throughput. The data have been deposited with links to BioProject accession number PRJNA509121 in the NCBI BioProject database (https://www.ncbi.nlm.nih.gov/bioproject/).

### Alignment of sequencing data

The quality of sequencing reads was evaluated using FastQC (Babraham Bioinformatics). As the quality of reads was judged to be high no additional filtering or trimming was performed. Reads were aligned to the human reference genome (hg38 NCBI - iGenomes) using Bowtie2 [38]. A 'local' alignment was performed on single end reads with the 'very-sensitive' pre-set parameters. Further processing of the alignment files was performed using samtools [39].

### Identifying differential expression

Reads mapping to known exons were counted using featureCounts [40] in an unstranded fashion and using the human genome annotation as a reference (hg38 NCBI – iGenomes). To characterise the variation between sequencing samples, edgeR [41] was used to produce a MDS plot. On the basis of the MDS plot two samples (one young baseline and one older ECC) were removed from the analysis. Differential expression was inferred between groups (i.e. young baseline versus young CON) using edgeR. In the edgeR analysis genes were only included in the analysis if the counts per million (CPM) for that gene was greater than 2 in more than 10 samples. Counts were normalised by library size and biological and technical variation (dispersion) was estimated. Significance of differential expression was estimated using the Fisher's exact test and *P*-values were corrected for a false discovery rate using the method of Benjamini and Hochberg [42] with a significance cut-off of *P-corr* < 0.05.

### Comparing differentially expressed genes across age and exercise modes

To identify overlapping differentially expressed genes we used the rank-rank hypergeometric overlap (RRHO) where genes were ranked on log fold change and *P*<0.05 defined a significant overlap [43]. The overlap between the exercise modes was identified by comparing each exercise mode against baseline within age groups. The overlap between young and older post-exercise responses was identified by comparing CON or ECC exercise modes across age groups. Sample specific differentially expressed genes were defined as those genes that showed statistically different ranks in RRHO and significant differential expression in that sample. Overlapping differentially expressed genes were defined as those genes that showed no significant difference in rank and were significantly differentially expressed in both samples.

### Pathway analysis

Significant differentially expressed genes were tested for functional enrichment using the GO [44] and BiNGO [45]. In this analysis the ‘sample’ was the set of differentially expressed genes and the ‘background’ was all genes that passed the filtering criteria in the edgeR analysis (see above). In particular, we tested for enrichment of GO Biological Process terms using the hypergeometric test and used all annotations for human genes and GO terms. Lists of differentially expressed genes were also subject to pathway enrichment analysis using the Enrichr web server [46,47], with specific focus placed on enrichment for pathway terms contained within the Kyoto Encyclopaedia of Genes and Genomes (KEGG) database [48]. In each case, terms with a Benjamini-Hochberg [42] corrected P < 0.05 were accepted as being enriched. Significantly enriched GO Biological Process terms were subsequently visualised as a network using BiNGO and Cytoscape [49].

### Ranking contraction-regulated genes

To rank the top 10 differentially expressed genes for each contraction mode and age group, up-regulated and down-regulated genes for each condition (versus baseline) were ranked separately based on their logarithmic fold-change in expression (log2 fold change [LFC]), and prioritised candidate targets then selected in each instance as the top 10 genes with the greatest LFC. Insight into the functional role(s) of each identified candidate target was subsequently assign using the UniProt database (uniprot.org).

## Supplementary Material

Supplementary Figure

Supplementary Table S1

Supplementary Table S2

Supplementary Table S3
