# Supplementary material for: The acute transcriptional response to resistance exercise: impact of age and contraction mode
Source: Aging (Albany NY). 2019 Apr 15;11(7):2111–26. doi: 10.18632/aging.101904 (PMC6503873; doi:10.18632/aging.101904)
Supplement: Supplementary Figure [file aging-11-101904-s001.pdf]

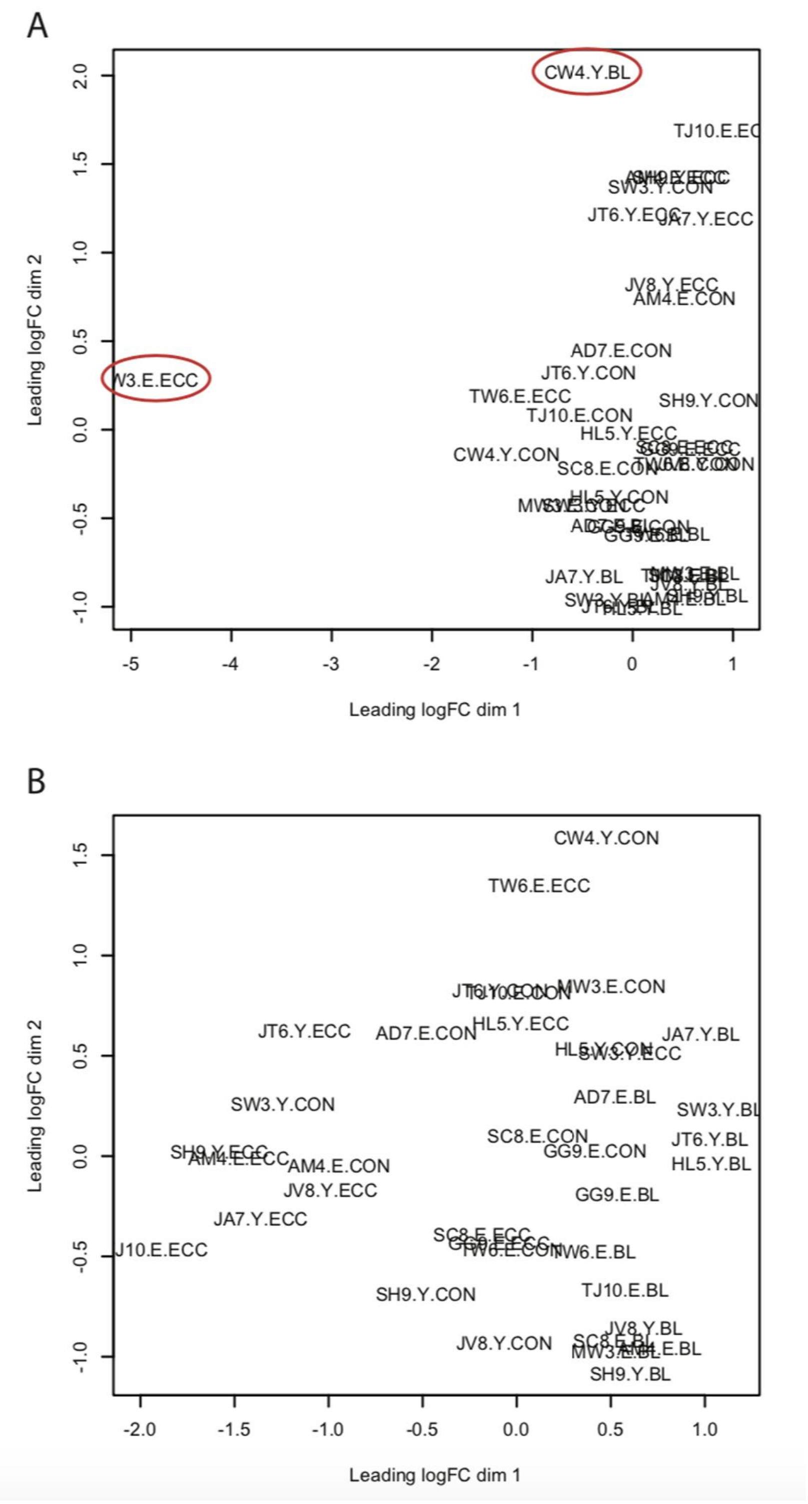

Figure S1. A multi-dimensional scaling (MDS) plot of the sequencing samples. (1A) Original MDS plot of all sequencing samples. From this plot two outliers were identified (circled in red) that did not cluster with the rest of the data. (1B) MDS of plot of remaining sequencing samples used for all analyses.
